# The Complexity of Singlehood: Desire for a Partner, Gender, and Age Differences

**DOI:** 10.1093/geroni/igaf122.2079

**Published:** 2025-12-31

**Authors:** Amy Rauer, Nadja Kelle, Oliver Huxhold

**Affiliations:** University of Tennessee, Knoxville, Tennessee, United States; German Centre of Gerontology, Berlin, Berlin, Germany; German Centre of Gerontology, Berlin, Berlin, Germany

## Abstract

Singlehood is often framed through societal stereotypes that portray singles as less happy or living a less fulfilled life than individuals in romantic relationships. However, this paper challenges such narratives by examining the diverse experiences of singlehood and the factors influencing satisfaction with being single. Using autoregressive models on longitudinal data from the German Aging Survey—a representative study of the German population aged 40 to 85—this study explores the role of the desire for a romantic partner, alongside gender and age, in shaping satisfaction with singlehood among the currently unpartnered. The findings reveal a significant negative association between the desire for a partner and satisfaction with singlehood across midlife and late adulthood. Furthermore, women and older individuals were less likely to express a desire for a partner than men and younger individuals. Interaction effects for gender and age did not moderate the association between desire for a partner and satisfaction with singlehood, suggesting a stable influence across demographics. Further analyses will incorporate relevant social factors, such as levels of social support and experiences of social exclusion, to investigate their role in satisfaction with singlehood. This study highlights the complexity of singlehood, showing that it is perceived negatively only when individuals desire a romantic partner—a desire less common among women and older individuals. This variation in partner-seeking helps explain differences in satisfaction with singlehood. The findings emphasize the need to challenge societal perceptions, advocating for a more nuanced and positive understanding of single life.

